# Alcohol consumption and cerebrospinal fluid biomarkers for preclinical alzheimer’s disease in a population-based sample of 70-year-olds

**DOI:** 10.1186/s13195-025-01819-2

**Published:** 2025-07-25

**Authors:** Silke Kern, Tobias Skillbäck, Henrik Zetterberg, Anna Dittrich, Felicia Ahlner, Anna Zettergren, Margda Waern, Nazib M Seidu, Ulf Andreasson, Kaj Blennow, Ingmar Skoog

**Affiliations:** 1 Department of Neuropsychiatric Epidemiology Unit, Institute of Neuroscience and Physiology, Sahlgrenska Academy at the University of Gothenburg, Gothenburg, Wallinsgatan 6, Mölndal, 43141 Sweden; 2 Clinical Neurochemistry Laboratory Department of Psychiatry and Neurochemistry, Institute of Neuroscience and Physiology, Institute of Neuroscience and Physiology, Sahlgrenska Academy at the University of Gothenburg, Gothenburg, Wallinsgatan 6, Mölndal, 43141 Sweden; 3 Department of Neuropsychiatry, Västra Götalandsregion, Sahlgrenska University Hospital, Gothenburg, Sweden; 4 Department of Neurodegenerative Disease, UCL Institute of Neurology, Queen Square, London, WC1N 3BG UK; 5 UK Dementia Research Institute at UCL, London, WC1N 3BG UK; 6 Hong Kong Center for Neurodegenerative Diseases, Hong Kong, China

**Keywords:** Alcohol consumption, Biomarkers, Cerebrospinal fluid, Alzheimer’s disease, Aβ42, P-tau, T-tau, NfL, Neurogranin

## Abstract

**Background:**

It is largely unknown how alcohol use affects the risk of Alzheimer`s disease (AD). Therefore, studies on the influence of alcohol use on cerebrospinal fluid (CSF) biomarkers for the earliest preclinical phase of AD are needed.

**Methods:**

This was a cross-sectional cohort study. The sample (*n* = 301) was derived from the 2014–2016 examinations of the Gothenburg H70 Birth Cohort Studies. The study cohort consisted of 301 70-year-old women and men, where of 246 cognitively unimpaired and 55 with mild cognitive deficits. Information on alcohol consumption (g/week and type of alcohol) was collected and CSF amyloid-β_1−42_ (Aβ42), total-tau (T-tau), tau phosphorylated at threonine 181 (P-tau181), neurofilament light protein (NfL) and neurogranin (Ng) were measured. We tested the association between the CSF biomarkers and alcohol consumption types using correlation and linear regression, adjusting for possible confounders when necessary according to the performed sensitivity analysis.

**Results:**

There were no correlations between weekly alcohol consumption and any of the CSF markers studied in the total sample of cognitively unimpaired participants (*n* = 246). After adjustments for multiplicity with FDR, there was an association between white wine and Ng in women with CDR = 0 (β:0.254, CI: ( 0.069: 0.439), *p* = 0.0076, FDR = 0.0455). Interaction analysis between female sex and red wine intake was a significant predictor of high Ng levels (β:0.410, CI: ( 0.099: 0.721), *p* = 0.0100, FDR = 0.0500). There were no correlations between consumption of specific types of alcohol (spirits, white wine, red wine, fortified wine, and beer) and any of the biomarkers studied in the total sample of cognitively unimpaired participants.

**Conclusions:**

Our findings indicate that higher alcohol use in older cognitively unimpaired women correlates with a biomarker of synaptic dysfunction in AD, which is an important observation in a time when alcohol use is increasing among women.

**Supplementary Information:**

The online version contains supplementary material available at 10.1186/s13195-025-01819-2.

## Introduction

The average life expectancy after age 65 years will increase worldwide from 18 to 20 years for women and from 16 to 18 for men until 2050 [1]. Sweden has an even higher life expectancy after age 65, with an expected increase from 22 to 24 years for women and 19 to 22 years for men [2]. This will result in a large increase in the number of older adults. In recent years it has been reported that newer generations of older people tend to drink more alcohol than previous generations [3]. Sex differences in patterns of alcohol consumption are also changing. Globally, men consume more alcohol and have higher rates of alcohol-related harm than women [4]. However, there is a diminishing gap between men and women in alcohol use in recent years [5–7]. With an increasing number of older people, it is important to understand if increased alcohol use will lead to an increase in brain diseases or dementia. Dementia is one of the leading causes of death and disability among older people. Although alcohol has an established neurotoxic effect [8], it is yet unclear if it is a risk factor or protective factor for the development of dementia, or if it has no effect. Epidemiological studies have given disparate results [9]. A meta-analysis reported that light to moderate alcohol use in middle to late adulthood was associated with a decreased risk of cognitive impairment and dementia. Heavy alcohol use was associated with changes in brain structures, cognitive impairments, and an increased risk for all types of dementia [9]. Previous studies suggest that not only alcohol per se, but also the type of alcohol may be important in the association between alcohol and neurodegenerative disorders. For example, wine, and especially red wine, has been suggested to be protective for dementia and spirits are suggested to be detrimental [10].

It is not clear if alcohol consumption in cognitively unimpaired individuals is related to Alzheimer’s disease (AD). We therefore sought to examine the relationship between the core cerebrospinal fluid (CSF) biomarkers of AD, amyloid-β_1−42_ (Aβ42), total-tau (T-tau), tau phosphorylated at threonine 181 (P-tau181), and alcohol consumption in a well-characterized population of older people derived from the Gothenburg H70 Birth Cohort Studies. We further included analysis of CSF biomarker and neurogranin (Ng), neurofilament light protein (NfL) in this context. Ng is a post-synaptic protein, that has been shown to be increased in AD as well at the presymptomatic stages of the disease [11]. NfL is an axonal protein that is released in response to general neurodegeneration, due to AD or other processes, and can be used to assess the amount of ongoing neuronal loss irrespective of cause [12].The overall aim of this study was to elucidate the potential link between alcohol intake and AD risk and development.

## Materials and methods

### Study cohort

The baseline sample was derived from the 2014–2016 examinations of the Gothenburg H70 Birth Cohort Studies in Gothenburg, Sweden. The sample was obtained from the Swedish Population Registry and included both persons living in private households and in residential care [13].

Every 70-year-old in Gothenburg, Sweden, born during 1944 on prespecified birth dates, was invited to the examination in 2014–2016, and 1203 participated (response rate 72.2%) [14]. Of these, 430 (35.8%) consented to a lumbar puncture. Contraindications (anticoagulant therapy, immune modulated therapy or cancer therapy) were present in 108, leaving 322 (26.8%). CSF volume was insufficient for two participants. Seven participants had dementia and were therefore excluded, while 55 had clinical dementia rating (CDR) 0.5. One participant had missing information on CSF neurofilament light protein (NfL) levels and total alcohol consumption, and five participants missed information on white wine, beer, depression, length of education and *APOE* status, leaving 246 participants with CDR 0 and 55 with CDR 0.5 for the present analyses.

### Study participant assessments and examinations

Participants were examined at the Neuropsychiatric Memory Clinic at Sahlgrenska University Hospital in Gothenburg or at home. Experienced psychiatric research nurses performed the *Neuropsychiatric examinations*, which comprised ratings of psychiatric symptoms and signs, and tests of mental functioning including assessments of episodic memory (short-term, long-term), aphasia, apraxia, agnosia, executive functioning and changes in selected personality traits according to participant and key informant interviews [15]. The Mini Mental State Examination was included as a marker of global cognitive function. Methods for key informant interviews have been previously described [15].

The CDR ratings were assigned by a geriatric psychiatrist and neurologist (SK). Dementia was diagnosed according to the DSM-III-R criteria [15].

Information on stroke and transient ischemic attack was acquired from self-reports, key-informants and the Swedish National Hospital Discharge Register. The participants underwent comprehensive somatic examinations [15]. Length of education was reported by self-report or close informant information.

### Alcohol consumption

Information regarding alcohol use was obtained by semi-structured face-to-face interviews performed by trained personnel. Average weekly amounts of beer, white wine, red wine, fortified wine and spirits consumed during the past month were reported. Frequency of beverage consumption was reported as ‘never’, ‘≤2 days/week’, ‘3–5 days/week’, or ‘>5 days/week’. Total alcohol consumption *(grams of pure alcohol per week)* was calculated using conversion factors based on average alcohol concentration by volume. We then split alcohol consumption into 3 groups. The first group comprised participants drinking 0 g/week alcohol, the second group of participants drinking 1–98 g/week (moderate alcohol consumption) and the third group consisted of participants drinking more than 98 g/week (risk drinkers) [16]. In addition, abstainers from alcohol were classified into two groups: “lifetime abstainers” (*n* = 3) and “previous consumers” (*n* = 10).

### Apolipoprotein E (*APOE*) genotyping

The SNPs rs7412 and rs429358 in *APOE* (gene map locus 19q13.2) were genotyped using KASPar^®^ PCR SNP genotyping system (LGC Genomics, Hoddesdon, Herts, UK). Genotype-data for these two SNPs were used to define ε2, ε3, and ε4 alleles.

### Cerebrospinal fluid sampling and biomarker analyses

Lumbar punctures (LP) to collect CSF samples were performed in the L3/L4 or L4/L5 inter-space in the morning [17]. The first 10 mL of CSF were collected in a polypropylene tube and immediately transported to the laboratory for centrifugation at 1800 g in 20^◦^C for 10 min. The supernatant was gently mixed to avoid possible gradient effects, aliquoted in polypropylene tubes and stored at − 70^◦^C [17, 18].

T-tau and P-tau181 were determined using a sandwich enzyme-linked-immunosorbent-assay (ELISA) (INNOTEST^®^ htau Ag and PHOSPHO_TAU (181P), Fujirebio, Ghent, Belgium) [19, 20]. CSF Aβ42 was measured using a sandwich ELISA (INNOTEST^®^ β-amyloid_1− 42_), specifically constructed to measure Aβ starting at amino acid 1 and ending at amino acid 42 [21]. For the Aβ42/Aβ40 ratio the V-PLEX Aβ Peptide Panel 1 (6E10) Kit (Meso Scale Discovery, Rockville, MD) was used [22]. CSF levels of NfL [23] and Ng [24] were analyzed using in-house ELISA methods. All assays are included in the panel of clinical routine analyses at the Clinical Neurochemistry Laboratory, Sahlgrenska University Hospital, Mölndal, Sweden. Analytic runs had to pass quality control criteria for the calibrators, and internal quality control samples had to be approved. This procedure has been described in more detail previously [25]. The CSF cut-offs in this study were: CSF Aβ42 levels ≤ 530 pg/mL, CSF T-tau levels ≥ 350 pg/mL and P-tau181 levels of ≥ 80 pg/mL [18].

### Statistical analyses

Characterizing summary statistics are presented in means (SD) and percentages (%) in Table 1. Differences in continuous variables (i.e., weekly consumption of pure alcohol) were tested with the Mann–Whitney *U*-test and differences in means were tested with T-tests, Fisher exact test was used for categorical variables. We applied linear regression models to test the association between the CSF biomarkers as dependent variables and alcohol consumption types as independent predicting variables, adjusting for additional possible confounders (education in years, depression, *APOE* status, smoking status, hypertension and diabetes) in the sample with CDR 0 (*n* = 246) as main analysis and in CDR 0.5 (*n* = 55) as secondary supplementary analysis. We performed sensitivity analysis excluding abstainers/stopped drinkers in the correlation analyses that were significant in CDR 0. Interactions between alcohol consumption and sex were examined with additional linear regression models. Interactions between total alcohol consumption and consumption of specific alcohol types (spirits, white wine, red wine, fortified wine and beer) were examined in supplementary analyses. Additional interaction between total alcohol consumption and types of alcohol consumption in the relation to the CSF biomarkers were tested in the total sample. Biomarker distributions were deemed sufficiently normally distributed for analysis in all instances, except for CSF NfL. Logarithmic transformation was applied to adjust for the skewed distribution of CSF NfL. Analyses were carried out in R studio 4.4.0 and IBM SPSS Statistics 24 for Windows (Armonk, NY). All *p*‐values were 2‐tailed, and *p*‐values < 0.05 were considered statistically significant. P-values for the models adjusted for possible confounders and interaction analyses were corrected for multiple comparison using the false discovery rate (FDR).

**Table 1:** Characteristics of the 301 participants with CSF and alcohol consumption data in the H70 Gothenburg birth cohort study of 70-year-olds

Variables	Total (*N* = 301)	CDR = 0 (*n* = 246)	CDR = 0.5 (*n* = 55)	*P*-value
Age in years Length of education in years MMSE score	70.91 (0.34) 12.75 (3.91) 28.94 (1.26)	70.91 (0.35) 13.07 (3.89) 29.22 (1.02)	70.91 (0.32) 11.36 (3.68) 27.65 (1.43)	0.95 **0.003** **< 0.001**
Women Living alone Any depression *APOE ε4* carriers Stroke Current smokers Hypertension Diabetes	142 (47.2%) 198 (65.8%) 28 (9.3%) 108 (35.9%) 6 (2%) 24 (8%) 181 (60.1%) 27 (9%)	120 (48.8%) 83 (33.7%) 20 (8.1%) 82 (33.3%) 4 (1.6%) 17 (6.9%) 145 (58.9%) 21 (8.6%)	22 (40.0%) 20 (36.4%) 8 (14.5%) 26 (47.3%) 2 (3.6%) 7 (12.7%) 36 (65.5%) 6 (10.9%)	0.3 0.8 0.2 0.06 **0.02** 0.2 0.4 0.6
CSF Aβ42, pg/ml CSF T-Tau, pg/ml CSF P-Tau181, pg/ml Log CSF NfL, pg/ml CSF Ng, pg/ml	721.55 (224.13) 331.65 (135.59) 49.21 (17.07) 6.59 (0.41) 204.51 (69.59)	728.54 (224.78) 331.99 (142.33) 49.20 (17.79) 6.60 (0.41) 205.88 (72.65)	690.29 (220.51) 330.11 (101.10) 49.27 (13.50) 6.57 (0.39) 198.39 (53.94)	0.25 0.91 0.97 0.64 0.39
Alcohol consumption, grams /week Lifetime abstainers Previous drinkers Alcohol per week < 1 g Alcohol per week 1–98 g Alcohol per week > 98 g	100.33 (148.24) 3(1%) 13 (4.32%) 35 (11.63%) 152 (50.5%) 98 (32.56%)	101.50 (156.50) 3 (1.2%) 10 (4.1%) 26 (10.6%) 128 (52%) 79 (32.1%)	95.09 (104.57) 0 3 (5.45%) 9 (16.36%) 24 (43.64%) 19 (34.55%)	0.71 1 0.71 0.25 0.30 0.75

Comparisons were performed with students t-test for continuous variables and Fisher’s exact test for categorical variables

Continuous variables are presented as mean (SD) and categorical variables as n (%). Abbreviations: Aβ42 = CSF amyloid-β1–42, T-tau = CSF total-tau, P-tau181 = CSF tau phosphorylated at threonine 181, NfL = CSF neurofilament light protein, Ng = CSF neurogranin

## Results

Baseline characteristics of the 301 participants are given in Table 1. Mean age was 70.91 (0.34) years, mean length of education was 12.75 (3.91) years, 47.2% (*n* = 142) were female, and 35.9% (*n* = 108) were *APOE* ε4 allele carriers. The study population contained 32.56% (*n* = 98) risk drinkers (> 98 g/week), 50.5% (*n* = 152) moderate drinkers (1–98 g/week) and 16.95% (*n* = 51) abstainers (< 1 g/week, lifetime abstainers and previous consumers). Participants with CDR 0 differed from those with CDR 0.5 in education, MMSE score and stroke (Table 1). In addition, participants with CDR 0 did not differ from participants with CDR 0.5 regarding CSF biomarkers and alcohol consumption. As previously reported, participants who donated CSF did not differ from those who did not in most respects, but history of stroke was less common in those with CSF due to contraindications for lumbar puncture [25]. The scatter plots between CSF biomarkers and total alcohol consumption in relation to sex and CDR group are shown in Supplementary Figure S1, S2 and S3.

In the group with CDR 0 (*n* = 246), there were no associations between total alcohol consumption or consumption of a specific alcohol type in grams per week and levels of any CSF biomarker (Table 2). When stratifying by sex (women, *n* = 120, men, *n* = 126), alcohol consumption did associate with several markers of neurodegeneration in women, but not in men. In men, there were no statistically significant associations between any type of alcohol intake and biomarkers of neurodegeneration (Table 2). In women, higher intake of beer was associated with lower levels of Aβ42 (β = -0.21, CI = -0.38 - -0.028), *p* = 0.024). High intake of red wine was associated with higher T-tau (β:0.204, CI: (0.018: 0.391), *p* = 0.0320) and P-tau181 levels (β:0.199, CI: (0.013: 0.385), *p* = 0.0364), while beer intake was associated with lower intake of T-tau (β:-0.195, CI: (-0.384: -0.007), *p* = 0.0425) (Table 2). There was no association between any type of alcohol intake and NfL, while higher intake of total alcohol (β:0.210, CI: ( 0.021: 0.398), *p* = 0.0295), white wine (β:0.254, CI: ( 0.069: 0.439), *p* = 0.0076) or red wine (β:0.299, CI: ( 0.041: 0.417), *p* = 0.0177) all were associated with higher levels of Ng. After adjustments for multiplicity with FDR, the association between white wine and Ng was the only one remaining significant (FDR = 0.0455) (Table 2). Total intake of alcohol did not interact significantly with intake of any specific alcohol form as predictors of biomarker levels with the exception of an interaction between beer intake and total alcohol intake for prediction of higher Ng-levels (β: 0.060, CI: 0.017: 0.103, FDR: 0.0170) (Supplementary table S1). Mean levels of CSF markers did not differ between risk drinkers, moderate drinkers and abstainers in neither univariate analyses, nor after controlling for age and education. When excluding abstainers (*n* = 8), a sensitivity correlation test between white wine consumption and higher Ng (*r* = 0.20, *p* = 0.04) and red wine consumption and higher Ng (*r* = 0.24, *p* = 0.013), higher T-tau (*r* = 0.23, *p* = 0.017) and P-tau181 (*r* = 0.22 *p* = 0.021) remained significant.

**Table 2:** Linear association between CSF biomarkers and alcohol consumption in participants with CDR = 0 (*n* = 246)

	All			Men			Women		
	β (CI)	*p*-value	FDR	β (CI)	*p*-value	FDR	β (CI)	*p*-value	FDR
**Aβ42**									
Total alcohol	0.042 (-0.082: 0.166)	0.5076	0.8574	0.081 (-0.096: 0.258)	0.3657	0.8751	-0.023 (-0.203: 0.157)	0.8009	0.8009
Spirit	0.025 (-0.099: 0.150)	0.6894	0.8574	0.029 (-0.152: 0.210)	0.7525	0.8751	0.082 (-0.092: 0.256)	0.3521	0.5281
White wine	-0.048 (-0.169: 0.072)	0.4288	0.8574	0.014 (-0.163: 0.191)	0.8751	0.8751	-0.116 (-0.292: 0.061)	0.1979	0.5281
Red wine	0.091 (-0.030: 0.212)	0.1386	0.8317	0.081 (-0.096: 0.257)	0.3660	0.8751	0.088 (-0.092: 0.267)	0.3348	0.5281
Fortified wine	-0.013 (-0.133: 0.106)	0.8266	0.8574	0.033 (-0.141: 0.207)	0.7092	0.8751	-0.053 (-0.225: 0.119)	0.5444	0.6533
Beer	0.011 (-0.113: 0.135)	0.8574	0.8574	0.046 (-0.134: 0.226)	0.6128	0.8751	-0.205 (-0.383: -0.027)	**0.0243**	0.1457
**T-tau**									
Total alcohol	0.024 (-0.106: 0.154)	0.7139	0.8812	0.011 (-0.168: 0.190)	0.9009	0.9961	0.121 (-0.068: 0.309)	0.2074	0.3111
Spirit	0.012 (-0.118: 0.142)	0.8537	0.8812	0.109 (-0.072: 0.290)	0.2371	0.9961	-0.062 (-0.247: 0.122)	0.5044	0.5044
White wine	0.057 (-0.069: 0.183)	0.3716	0.8812	0.082 (-0.096: 0.259)	0.3650	0.9961	0.125 (-0.061: 0.312)	0.1867	0.3111
Red wine	0.062 (-0.065: 0.188)	0.3390	0.8812	0.000 (-0.178: 0.179)	0.9961	0.9961	0.204 ( 0.018: 0.391)	**0.0320**	0.1275
Fortified wine	-0.009 (-0.134: 0.115)	0.8812	0.8812	-0.018 (-0.193: 0.158)	0.8403	0.9961	-0.063 (-0.245: 0.119)	0.4943	0.5044
Beer	-0.032 (-0.161: 0.097)	0.6260	0.8812	-0.057 (-0.238: 0.124)	0.5334	0.9961	-0.195 (-0.384: -0.007)	**0.0425**	0.1275
**P-tau181**									
Total alcohol	0.016 (-0.113: 0.145)	0.8058	0.8486	0.013 (-0.163: 0.190)	0.8809	0.9691	0.084 (-0.105: 0.273)	0.3788	0.5682
Spirit	0.013 (-0.117: 0.142)	0.8486	0.8486	0.105 (-0.074: 0.283)	0.2485	0.9691	-0.045 (-0.229: 0.139)	0.6265	0.6265
White wine	0.018 (-0.107: 0.143)	0.7750	0.8486	0.072 (-0.103: 0.247)	0.4176	0.9691	0.057 (-0.131: 0.244)	0.5509	0.6265
Red wine	0.062 (-0.064: 0.188)	0.3355	0.8486	0.003 (-0.173: 0.180)	0.9691	0.9691	0.199 ( 0.013: 0.385)	**0.0364**	0.1943
Fortified wine	-0.022 (-0.147: 0.102)	0.7215	0.8486	-0.027 (-0.200: 0.146)	0.7564	0.9691	-0.082 (-0.263: 0.099)	0.3723	0.5682
Beer	-0.025 (-0.153: 0.104)	0.7048	0.8486	-0.049 (-0.228: 0.130)	0.5876	0.9691	-0.178 (-0.366: 0.011)	0.0648	0.1943
**NfL**									
Total alcohol	0.022 (-0.109: 0.153)	0.7404	0.7404	0.036 (-0.144: 0.216)	0.6901	0.9902	-0.028 (-0.225: 0.169)	0.7795	0.9517
Spirit	-0.044 (-0.175: 0.087)	0.5072	0.7404	0.001 (-0.182: 0.185)	0.9902	0.9902	-0.148 (-0.337: 0.042)	0.1257	0.3836
White wine	0.028 (-0.098: 0.155)	0.6609	0.7404	0.026 (-0.154: 0.205)	0.7768	0.9902	0.039 (-0.156: 0.234)	0.6947	0.9517
Red wine	-0.025 (-0.152: 0.103)	0.7056	0.7404	-0.064 (-0.243: 0.116)	0.4831	0.9902	0.026 (-0.171: 0.224)	0.7931	0.9517
Fortified wine	-0.073 (-0.198: 0.052)	0.2505	0.7404	0.003 (-0.174: 0.180)	0.9729	0.9902	-0.145 (-0.332: 0.042)	0.1279	0.3836
Beer	0.048 (-0.082: 0.178)	0.4655	0.7404	0.063 (-0.119: 0.245)	0.4960	0.9902	-0.004 (-0.204: 0.195)	0.9663	0.9663
**Ng**									
Total alcohol	0.055 (-0.077: 0.187)	0.4138	0.6207	0.014 (-0.169: 0.196)	0.8835	0.9421	0.210 ( 0.021: 0.398)	**0.0295**	0.0590
Spirit	-0.070 (-0.202: 0.062)	0.2989	0.5978	-0.024 (-0.210: 0.162)	0.7971	0.9421	-0.059 (-0.246: 0.129)	0.5366	0.5366
White wine	0.093 (-0.035: 0.221)	0.1525	0.4576	0.012 (-0.170: 0.193)	0.8973	0.9421	0.254 ( 0.069: 0.439)	**0.0076**	**0.0455**
Red wine	0.099 (-0.030: 0.228)	0.1308	0.4576	0.027 (-0.154: 0.209)	0.7656	0.9421	0.229 ( 0.041: 0.417)	**0.0177**	0.0531
Fortified wine	-0.008 (-0.135: 0.120)	0.9072	0.9072	0.050 (-0.128: 0.229)	0.5766	0.9421	-0.098 (-0.282: 0.086)	0.2924	0.3509
Beer	0.013 (-0.119: 0.145)	0.8437	0.9072	0.007 (-0.178: 0.192)	0.9421	0.9421	-0.175 (-0.367: 0.017)	0.0736	0.1104

Due to the observed differences between men and women in the correlation analysis interactions between alcohol consumption and sex were examined in additional age-adjusted linear models (Table 3). There was an interaction between female sex and white wine intake for lower Aβ42 levels (β = -0.26, CI = -0.51 to -0.006, *p* = 0.045). Intake of red wine intake interacted with female sex for higher levels of T-tau (β = 0.39, CI = 0.077 to 0.70), *p* = 0.0149), P-Tau181 (β = 0.37, CI = 0.054 to 0.68, *p* = 0.022) and Ng (β = 0.41, CI = 0.099 to 0.72, *p* = 0.010) (Table 3). After adjustment for multiplicity, the only remaining borderline significant association was between high intake of red wine in women and increased Ng (FDR = 0.05).

**Table 3:** Interaction analyses between alcohol consumption type and sex, in relation to CSF biomarkers among participants with CDR = 0 (*n* = 246)

	β (CI)	*p*-value	FDR
**Aβ42**			
Total alcohol	-0.303 (-0.692: 0.085)	0.1257	0.2332
Spirit	0.082 (-0.271: 0.435)	0.6493	0.9078
White wine	-0.256 (-0.507: -0.006)	**0.0449**	0.2247
Red wine	-0.057 (-0.372: 0.258)	0.7217	0.9021
Fortified wine	-0.064 (-0.320: 0.192)	0.6245	0.8447
Beer	-0.893 (-2.540: 0.754)	0.2867	0.7168
**T-tau**			
Total alcohol	0.298 (-0.092: 0.689)	0.1338	0.6690
Spirit	-0.047 (-0.401: 0.308)	0.7957	0.7957
White wine	0.109 (-0.145: 0.362)	0.3988	0.8900
Red wine	0.391 ( 0.077: 0.704)	**0.0149**	0.0745
Fortified wine	-0.061 (-0.318: 0.196)	0.6397	0.8917
Beer	-1.570 (-3.216: 0.076)	0.0615	0.1536
**P-tau181**			
Total alcohol	0.221 (-0.167: 0.610)	0.2629	0.6572
Spirit	-0.011 (-0.363: 0.341)	0.9513	0.9513
White wine	0.055 (-0.197: 0.307)	0.6673	0.8719
Red wine	0.366 ( 0.054: 0.678)	**0.0215**	0.1077
Fortified wine	-0.077 (-0.332: 0.178)	0.5543	0.8652
Beer	-1.525 (-3.159: 0.109)	0.0672	0.1379
**NfL**			
Total alcohol	0.022 (-0.363: 0.407)	0.9096	0.9606
Spirit	-0.247 (-0.593: 0.099)	0.1605	0.2006
White wine	0.069 (-0.180: 0.318)	0.5855	0.7319
Red wine	0.177 (-0.134: 0.488)	0.2627	0.3284
Fortified wine	-0.074 (-0.326: 0.177)	0.5604	0.7005
Beer	0.098 (-1.529: 1.725)	0.9055	0.9055
**Ng**			
Total alcohol	0.446 ( 0.060: 0.832)	0.0237	0.1186
Spirit	0.041 (-0.312: 0.394)	0.8182	0.8182
White wine	0.235 (-0.015: 0.485)	0.0651	0.3254
Red wine	0.410 ( 0.099: 0.721)	**0.0100**	**0.0500**
Fortified wine	-0.153 (-0.408: 0.103)	0.2397	0.3995
Beer	-1.570 (-3.210: 0.070)	0.0606	0.3029

Women are set as norm and the regression is adjusted for age

Abbreviations: CI - confidence interval, FDR - false discovery rate, NfL - neurofilament light protein

In participants with CDR 0.5 (*n* = 55), there was an association between total alcohol consumption and CSF Ng levels (β = 0.31, CI = 0.016 to 0.60), *p* = 0.040)(Table 4). Sensitivity correlation analysis excluding two abstainers validated this observation (*r* = 0.30, *p* = 0.050). When stratifying by sex, among men, there was an association between higher total alcohol consumption and higher CSF T-tau (β = 0.49, CI = 0.11 to 0.87, *p* = 0.014, FDR = 0.045) and higher CSF Ng (β = 0.50, CI = 0.15 to 0.85), *p* = 0.0066, FDR = 0.037)(Table 4). There were no other associations in the CDR 0.5 group. Due to the limited sample with CDR 0.5 sub-analyses of beverage types were not feasible.

**Table 4:** Linear association between CSF biomarkers and alcohol consumption, in participants with CDR 0.5 (*n* = 55)

	All			Men			Women		
	β (CI)	*p*-value	FDR	β (CI)	*p-*value	FDR	β (CI)	*p*-value	FDR
Aβ42	0.000 (-0.272: 0.272)	0.9996	0.9996	0.047 (-0.327: 0.422)	0.7962	0.8791	-0.094 (-0.670: 0.482)	0.7298	0.8139
T-tau	0.249 (-0.044: 0.542)	0.0940	0.2819	0.489 ( 0.110: 0.868)	**0.0136**	**0.0453**	-0.400 (-0.965: 0.165)	0.1504	0.3008
P-tau181	0.117 (-0.180: 0.415)	0.4308	0.9804	0.287 (-0.118: 0.692)	0.1571	0.4038	-0.392 (-0.947: 0.163)	0.1512	0.3024
NfL	-0.126 (-0.413: 0.161)	0.3812	0.5717	-0.024 (-0.443: 0.395)	0.9085	0.9801	-0.436 (-0.872: 0.001)	0.0504	0.1511
Ng	0.309 ( 0.016: 0.601)	**0.0391**	0.2344	0.501 ( 0.153: 0.849)	**0.0066**	**0.0372**	-0.312 (-0.935: 0.311)	0.2992	0.5983

Models were adjusted for sex, age, education, depression, *APOE* status, smoking status, hypertension and diabetes

Abbreviations: CI - confidence interval, FDR - false discovery rate, NfL - neurofilament light protein

Interaction analyses in CDR 0.5 (*n* = 55) revealed interaction effects between sex and white wine consumption (β = -0.670, FDR = 0.0425) and sex and fortified wine consumption (β = -1.636, FDR = 0.03820.038) and Aβ42, as well as sex and total alcohol consumption (β = -0.872, FDR = 0.0305) and sex and white wine consumption (β = -0.963, FDR = 0.0019) in relation Ng after adjustment for multiplicity (Supplementary Table S2). In addition, we found an interaction effect between total alcohol consumption and fortified wine consumption in relation to CSF Aβ42, total alcohol consumption and fortified wine consumption in relation to CSF NfL, but none of these remained after correction for multiple testing (Supplementary Table S3).

## Discussion

In a population-based sample of 70-year-olds, we found sex-dependent associations between alcohol intake and markers of neurodegeneration in individuals with normal cognition and MCI. Specifically, we found associations between higher alcohol consumption and higher CSF Ng, a marker of synaptic degeneration, in cognitively healthy women, and in men with mild cognitive symptoms. The associations between higher alcohol consumption and higher CSF Ng remained also after excluding abstainers. Regarding beverage type, increased consumption of red wine was related to higher CSF Ng, as well as higher T-tau and P-tau181 levels, in women. While these associations did not hold for FDR-adjustment, the interaction between female sex and red wine consumption to predict Ng levels was still significant. Furthermore, high white wine consumption was still a significant predictor of Ng concentrations in women after FDR-adjustment. Women with high beer consumption also presented with lower levels of Aβ42 and T-tau levels, although this observation did not remain after FDR-adjustment. We have previously reported that risk drinking has increased dramatically in older people in Sweden, especially among women [3, 6]. Our findings raise concerns regarding the adverse health effects of alcohol consumption in this age group, especially among women, and in men with mild cognitive symptoms.

Our finding of a relation between higher alcohol consumption and higher Ng levels in women with CDR 0 is interesting as Ng is a CSF marker of synaptic dysfunction in AD. Studies in rodents and autopsy studies in patients with a history of severe alcohol consumption have shown that although all types of cells in the brain are vulnerable to the toxic effect of alcohol, it seems that synaptic terminals are major targets for alcohol toxicity, accounting for synaptic impairments [26]. Taken together, findings suggest that in older people, higher alcohol consumption may be related to synaptic dysfunction. It needs to be emphasized that the number of risk drinkers in this population-based study was small, and the relationship between higher alcohol consumption and synaptic dysfunction was not driven by risk drinkers. Our finding is notable as increased levels of CSF Ng have been shown already at the predementia stages [27–30]. It has been suggested that increased levels of Ng are specific for AD, as there are no changes in CSF levels of this marker in other degenerative disorders [31]. Our findings suggest that Ng may also be a marker for brain changes related to alcohol consumption. Alcohol consumption may increase risk for dementia in several ways. It may lead to general brain atrophy, especially in frontal areas of the brain, but it could also influence AD pathology specifically through oxidative stress, synaptic dysfunction, impaired amyloid clearance, tau phosphorylation or APOE4 interactions. Finally, alcohol use may lead to vascular changes in the brain, which may compromise cognitive function. However, it seems as well that the type of beverage consumed may play a role in the association between alcohol and neurodegenerative disorders. For example, wine, and especially red wine, has been suggested to be protective for dementia and spirits are suggested to be detrimental due to the cardioprotective effect of anti-inflammatory polyphenolic and bioactive substances including lipophilic molecules [10, 32]. Results on the possible protective effect of wine on dementia are mixed [10, 33]. One study among US community-dwelling participants (aged 72 years and older) found that higher alcohol consumption was related to lower risk for dementia among participants without mild cognitive impairment (MCI), and to higher risk among those with MCI [34]. Several studies report that moderate consumption of wine decreases the risk and high consumption of spirits is related to increased risk [10, 35–37]. It must be emphasized that our subgroups were small, and our findings need to be replicated in larger samples.

CSF Ng, T-tau and P-tau181 are AD biomarkers that change early in the disease course (already in the preclinical stage) [28, 29]. Interestingly, we found no association between consumption of alcohol and CSF NfL, a biomarker for general neurodegeneration, that changes later than Ng, T-tau and P-tau181 and is not specific for AD [30, 38, 39]. It is possible that the associations seen between alcohol and AD biomarkers will be followed by similar correlations for NfL. However, longitudinal samples are needed to address this hypothesis.

It must be emphasized that we did not see any linear relationship between overall alcohol consumption and any of the CSF biomarkers in a dose-response matter without stratification for sex after FDR-adjustments. This may be due to the small sample size or that the CSF biomarkers were derived from neurologically healthy older adults from the general population, or that alcohol consumption is not an important determinant of CSF markers in the population.

Studies examining alcohol consumption in relation to traditional CSF biomarkers of AD are rare [40–42] and none have, to our knowledge, examined alcohol consumption in relation to new markers such as NfL and Ng. In addition, most other studies on the relation between alcohol consumption and CSF markers are conducted in clinical samples on people with problematic drinking, while our study is community-based. We found no association between higher total alcohol consumption and levels of CSF Aβ42, T-tau or P-tau181 (and thus no associations with AD-type pathophysiology). This is in line with a cross-sectional multicenter study from pooled samples of older persons (mean age 70 years) with mild cognitive impairment or subjective cognitive decline recruited from memory clinics or the community from Finland, Netherlands and Sweden [41]. Another study from a memory clinic reported that CSF tau levels, but not CSF Aβ42, were higher in alcohol-related cognitive decline than in normal aging [43].

Our finding that red wine consumption was associated with higher CSF Ng, T-tau and P-tau181 in women as well as white wine and Ng is interesting due to the steep increase in wine consumption in women during the last decades [3]. Most of women’s consumption of alcohol was wine, and female consumption of beer and spirits was very low [3]. However, the sample was small, and findings should be interpreted cautiously. Our findings of subtle changes in women only support the view that women have lower tolerance for alcohol, which is reflected in national and international health recommendations.

### Strength and limitations

A major strength of this study was the well-characterized population with extensive neuropsychiatric and somatic examinations. However, this study had several limitations. First, although our CSF sample was large for a population-based study, it was small in overall size, (especially the CDR = 0.5 group), giving rise to low statistical power and the possibility for false negative findings. Second, alcohol consumption was ascertained by interview only and we had no objective measure of recent alcohol consumption (phosphatidylethanol or carbohydrate-deficient transferrin). Third, we lack data on lifetime alcohol consumption, and, since this was a cross-sectional study, firm conclusions on the causality between alcohol consumption and neurodegeneration cannot be drawn. Fourth, there was a large sample loss in selection (from *n* = 1203 to *n* = 322), mainly due to subjects not consenting to lumbar puncture, introducing a risk of selection bias. Fifth, while the study has made an effort to ask participants specifically on different types of beverages consumed, the sample size is not sufficient to fully account for all differences in consumption habits. Thus adjustments for multiple testing resulted in the mitigation of several observed associations. Independent studies with predetermined outcomes could validate our exploratory observations.

## Conclusions

We found associations between higher alcohol consumption and Ng in cognitively healthy women and in men with mild cognitive symptoms. It may be that women are especially vulnerable to alcohol in the early predementia stages. However, these findings should be taken cautiously due to small sample sizes.

## Electronic supplementary material

Below is the link to the electronic supplementary material.

Supplementary Material 1

Supplementary Material 2

Supplementary Material 3

Supplementary Material 4

## Data Availability

No datasets were generated or analysed during the current study.
