# Supplementary figures and images for: Alcohol consumption and cerebrospinal fluid biomarkers for preclinical alzheimer’s disease in a population-based sample of 70-year-olds
Source: Alzheimers Res Ther. 2025 Jul 25;17:175. doi: 10.1186/s13195-025-01819-2 (PMC12291311; doi:10.1186/s13195-025-01819-2)

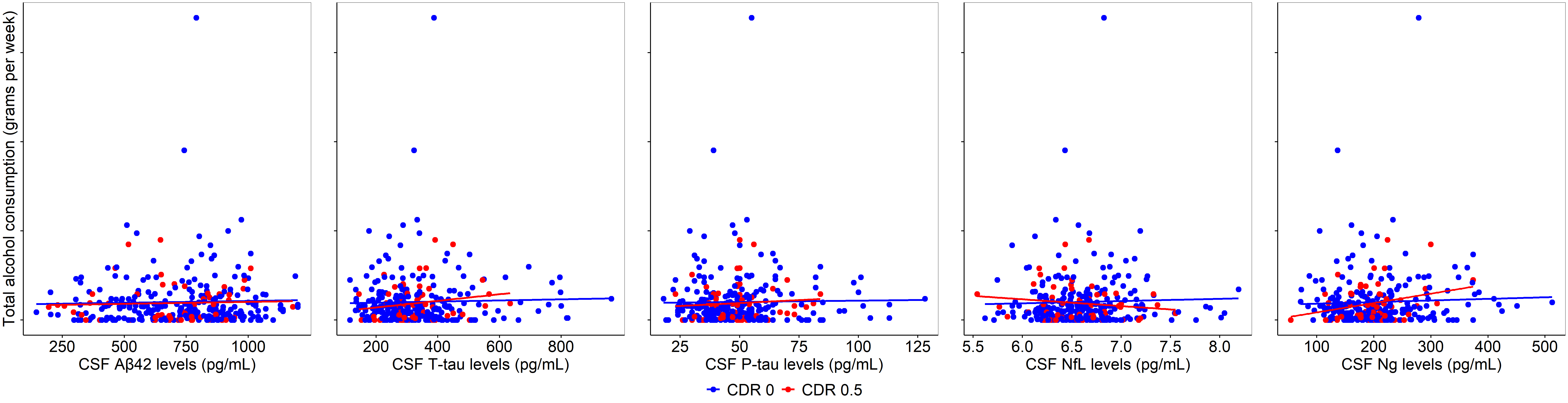

Supplement: Supplementary file 1 — Supplementary Material 1 [file 13195_2025_1819_MOESM1_ESM.png]

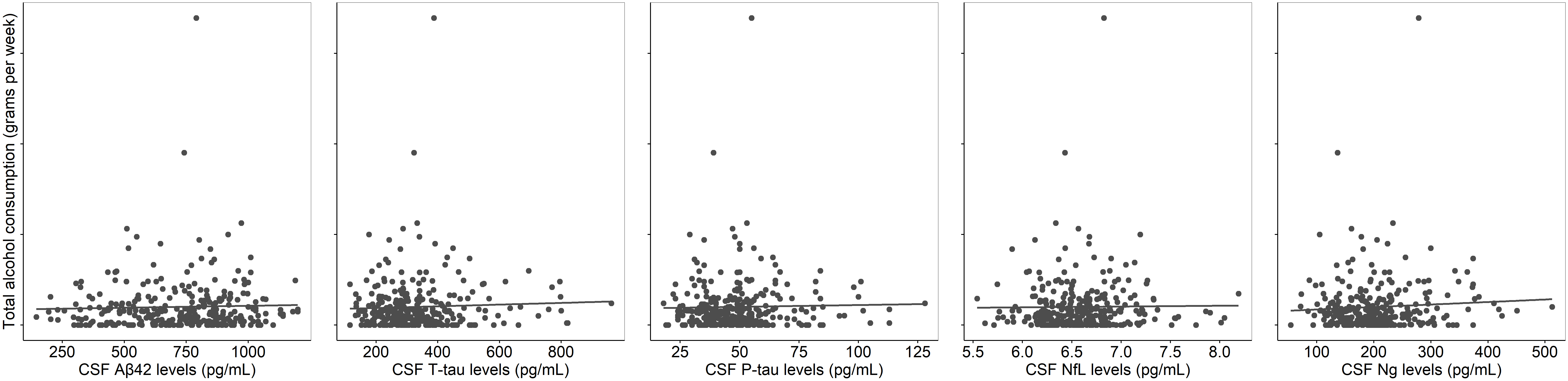

Supplement: Supplementary file 2 — Supplementary Material 2 [file 13195_2025_1819_MOESM2_ESM.png]

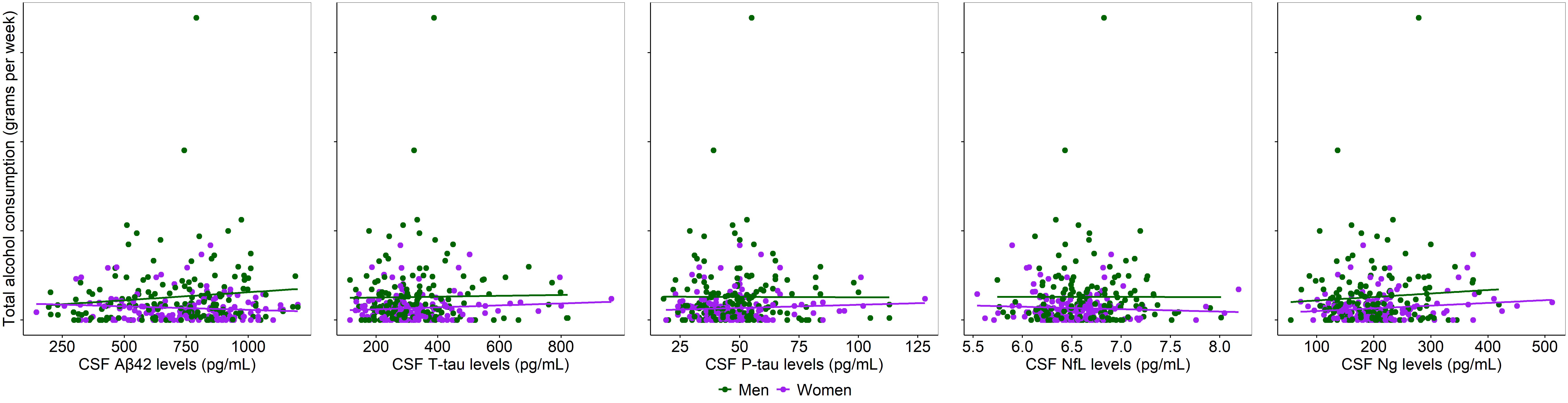

Supplement: Supplementary file 3 — Supplementary Material 3 [file 13195_2025_1819_MOESM3_ESM.png]
