# Supplementary material for: Alcohol consumption and cerebrospinal fluid biomarkers for preclinical alzheimer’s disease in a population-based sample of 70-year-olds
Source: Alzheimers Res Ther. 2025 Jul 25;17:175. doi: 10.1186/s13195-025-01819-2 (PMC12291311; doi:10.1186/s13195-025-01819-2)
Supplement: Supplementary file 4 — Supplementary Material 4 [file 13195_2025_1819_MOESM4_ESM.docx]

**SUPPLEMENTARY MATERIALS**

**Title**: Alcohol consumption and cerebrospinal fluid biomarkers for preclinical Alzheimer’s disease in a population-based sample of 70-year-olds

**Running title**: Alcohol consumption and CSF biomarkers of AD.

CONTENTS

Table S1: CDR 0: Interaction between total alcohol consumption and alcohol consumption types in relation to CSF biomarkers ...................................................................................................................................................... 2

Table S2: CDR 0.5: Interaction between alcohol consumption types and sex in relation to CSF biomarkers ….. 3

Table S3: CDR 0.5: Interaction between total alcohol consumption and alcohol consumption types in relation to CSF biomarkers ...................................................................................................................................................... 4

**Table S1: CDR 0: Interaction between total alcohol consumption and alcohol consumption types in relation to CSF biomarkers.**

| **CSF Biomarker** | **Interaction term** | **Beta** | **LCI** | **UCI** | **SE** | **P** | **FDR** |
| --- | --- | --- | --- | --- | --- | --- | --- |
| Amyloid-beta  Amyloid-beta  Amyloid-beta  Amyloid-beta  Amyloid-beta | Total alcohol:Spirit  Total alcohol:White wine  Total alcohol:Red wine  Total alcohol:Fortified wine  Total alcohol:Beer | -0.009  0.020  -0.018  0.064  -0.001 | -0.074  -0.077  -0.125  -0.224  -0.045 | 0.056  0.118  0.089  0.352  0.043 | 0.033  0.049  0.054  0.146  0.022 | 0.7842  0.6777  0.7405  0.6604  0.9707 | 0.9465  0.8963  0.9986  0.9802  0.9929 |
| T-tau  T-tau  T-tau  T-tau  T-tau | Total alcohol:Spirit  Total alcohol:White wine  Total alcohol:Red wine  Total alcohol:Fortified wine  Total alcohol:Beer | -0.030  -0.017  -0.037  0.045  0.041 | -0.094  -0.115  -0.145  -0.243  -0.003 | 0.035  0.080  0.070  0.333  0.084 | 0.033  0.049  0.055  0.146  0.022 | 0.3648  0.7241  0.4960  0.7599  0.0662 | 0.9120  0.9306  0.9967  0.9498  0.1656 |
| P-tau  P-tau  P-tau  P-tau  P-tau | Total alcohol:Spirit  Total alcohol:White wine  Total alcohol:Red wine  Total alcohol:Fortified wine  Total alcohol:Beer | -0.042  -0.028  -0.035  0.018  0.036 | -0.106  -0.125  -0.142  -0.267  -0.008 | 0.022  0.069  0.071  0.303  0.079 | 0.032  0.049  0.054  0.145  0.022 | 0.1959  0.5679  0.5158  0.9015  0.1068 | 0.3460  0.9122  0.8212  0.9940  0.1780 |
| NfL  NfL  NfL  NfL  NfL | Total alcohol:Spirit  Total alcohol:White wine  Total alcohol:Red wine  Total alcohol:Fortified wine  Total alcohol:Beer | -0.019  -0.053  -0.098  -0.027  -0.013 | -0.083  -0.150  -0.204  -0.312  -0.057 | 0.045  0.044  0.008  0.258  0.031 | 0.033  0.049  0.054  0.145  0.022 | 0.5638  0.2807  0.0709  0.8527  0.5560 | 0.9183  0.7017  0.1772  0.9911  0.8866 |
| Ng  Ng  Ng  Ng  Ng | Total alcohol:Spirit  Total alcohol:White wine  Total alcohol:Red wine  Total alcohol:Fortified wine  Total alcohol:Beer | -0.022  -0.081  -0.063  0.171  0.060 | -0.086  -0.177  -0.170  -0.116  0.017 | 0.042  0.016  0.043  0.457  0.103 | 0.033  0.049  0.054  0.145  0.022 | 0.5027  0.1001  0.2428  0.2415  0.0068 | 0.8378  0.2502  0.4046  0.4679  0.0170 |

LCI = lower confidence limit, UCI = upper confidence limit, SE = standard error, P = p-value and FDR = false discovery rate. Models were adjusted for age.

**Table S2: CDR 0.5: Interaction between alcohol consumption types and sex in relation to CSF biomarkers.**

| **CSF Biomarker** | **Interaction term** | **Beta** | **LCI** | **UCI** | **SE** | **P** | **FDR** |
| --- | --- | --- | --- | --- | --- | --- | --- |
| Aymloid-beta  Aymloid-beta  Aymloid-beta  Aymloid-beta  Aymloid-beta  Aymloid-beta | Sex (Women):Total alcohol  Sex (Women):Spirit  Sex (Women):White wine  Sex (Women):Red wine  Sex (Women):Fortified wine  Sex (Women):Beer | -0.323  2.716  -0.670  -0.170  -1.636  -0.533 | -1.071  -1.157  -1.216  -0.779  -2.944  -1.809 | 0.425  6.588  -0.125  0.438  -0.328  0.743 | 0.372  1.928  0.271  0.303  0.651  0.635 | 0.3904  0.1651  0.0170  0.5763  0.0153  0.4058 | 0.9363  0.7410  0.0425  0.9467  0.0382  0.7465 |
| T-tau  T-tau  T-tau  T-tau  T-tau  T-tau | Sex (Women):Total alcohol  Sex (Women):Spirit  Sex (Women):White wine  Sex (Women):Red wine  Sex (Women):Fortified wine  Sex (Women):Beer | -0.793  -1.947  -0.713  -0.339  0.496  -1.032 | -1.492  -5.830  -1.259  -0.949  -0.879  -2.254 | -0.094  1.937  -0.166  0.270  1.871  0.190 | 0.348  1.934  0.272  0.304  0.685  0.608 | 0.0269  0.3189  0.0116  0.2687  0.4722  0.0961 | 0.0672  0.7120  0.0582  0.7491  0.5530  0.2402 |
| P-tau  P-tau  P-tau  P-tau  P-tau  P-tau | Sex (Women):Total alcohol  Sex (Women):Spirit  Sex (Women):White wine  Sex (Women):Red wine  Sex (Women):Fortified wine  Sex (Women):Beer | -0.694  -1.582  -0.657  -0.325  0.510  -1.154 | -1.422  -5.516  -1.213  -0.940  -0.878  -2.400 | 0.033  2.352  -0.101  0.289  1.898  0.091 | 0.362  1.958  0.277  0.306  0.691  0.620 | 0.0608  0.4231  0.0215  0.2927  0.4639  0.0686 | 0.3040  0.9987  0.1073  0.9596  0.9916  0.3430 |
| NfL  NfL  NfL  NfL  NfL  NfL | Sex (Women):Total alcohol  Sex (Women):Spirit  Sex (Women):White wine  Sex (Women):Red wine  Sex (Women):Fortified wine  Sex (Women):Beer | -0.524  -0.588  -0.214  -0.161  1.492  -0.764 | -1.231  -4.398  -0.753  -0.745  0.241  -1.992 | 0.182  3.222  0.326  0.423  2.744  0.465 | 0.352  1.897  0.268  0.291  0.623  0.612 | 0.1424  0.7579  0.4300  0.5817  0.0204  0.2174 | 0.3559  0.9599  0.7166  0.8042  0.0510  0.5436 |
| Ng  Ng  Ng  Ng  Ng  Ng | Sex (Women):Total alcohol  Sex (Women):Spirit  Sex (Women):White wine  Sex (Women):Red wine  Sex (Women):Fortified wine  Sex (Women):Beer | -0.872  -1.504  -0.963  -0.513  -0.283  -1.105 | -1.546  -5.416  -1.471  -1.094  -1.669  -2.329 | -0.199  2.407  -0.455  0.068  1.103  0.120 | 0.335  1.947  0.253  0.289  0.690  0.610 | **0.0122**  0.4435  0.0004  0.0820  0.6835  0.0761 | **0.0305**  0.7793  0.0019  0.2051  0.9722  0.1901 |

LCI = lower confidence limit, UCI = upper confidence limit, SE = standard error, P = p-value and FDR = false discovery rate. Models were adjusted for age.

**Table S3:** **CDR 0.5: Interaction between total alcohol consumption and alcohol consumption types in relation to CSF biomarkers.**

| **CSF Biomarker** | **Interaction term** | **Beta** | **LCI** | **UCI** | **SE** | **P** | **FDR** |
| --- | --- | --- | --- | --- | --- | --- | --- |
| Amyloid-beta  Amyloid-beta  Amyloid-beta  Amyloid-beta  Amyloid-beta | Total alcohol:Spirit  Total alcohol:White wine  Total alcohol:Red wine  Total alcohol:Fortified wine  Total alcohol:Beer | -0.151  0.273  -0.232  -1.250  0.039 | -0.586  -0.203  -0.561  -2.336  -0.188 | 0.284  0.750  0.097  -0.164  0.267 | 0.217  0.237  0.164  0.541  0.113 | 0.4889  0.2551  0.1630  0.0249  0.7288 | 0.8214  0.7383  0.4075  0.0623  0.8777 |
| T-tau  T-tau  T-tau  T-tau  T-tau | Total alcohol:Spirit  Total alcohol:White wine  Total alcohol:Red wine  Total alcohol:Fortified wine  Total alcohol:Beer | 0.209  -0.072  0.068  0.329  -0.004 | -0.216  -0.550  -0.266  -0.785  -0.229 | 0.633  0.407  0.401  1.442  0.220 | 0.211  0.238  0.166  0.554  0.112 | 0.3280  0.7643  0.6857  0.5559  0.9688 | 0.5144  0.9464  0.8134  0.6949  0.9862 |
| P-tau  P-tau  P-tau  P-tau  P-tau | Total alcohol:Spirit  Total alcohol:White wine  Total alcohol:Red wine  Total alcohol:Fortified wine  Total alcohol:Beer | 0.028  -0.174  -0.020  0.266  -0.064 | -0.409  -0.660  -0.361  -0.875  -0.293 | 0.464  0.312  0.321  1.407  0.165 | 0.217  0.242  0.170  0.568  0.114 | 0.8991  0.4756  0.9054  0.6417  0.5773 | 0.9353  0.8914  0.9448  0.8198  0.9392 |
| NfL  NfL  NfL  NfL  NfL | Total alcohol:Spirit  Total alcohol:White wine  Total alcohol:Red wine  Total alcohol:Fortified wine  Total alcohol:Beer | 0.134  -0.001  -0.018  1.112  -0.137 | -0.283  -0.455  -0.341  0.077  -0.353 | 0.550  0.453  0.306  2.148  0.079 | 0.207  0.226  0.161  0.516  0.108 | 0.5224  0.9964  0.9135  0.0358  0.2078 | 0.6817  0.9981  0.9784  0.0894  0.2597 |
| Ng  Ng  Ng  Ng  Ng | Total alcohol:Spirit  Total alcohol:White wine  Total alcohol:Red wine  Total alcohol:Fortified wine  Total alcohol:Beer | 0.175  0.132  0.032  -0.321  0.133 | -0.239  -0.334  -0.294  -1.415  -0.084 | 0.588  0.599  0.358  0.773  0.351 | 0.206  0.232  0.162  0.545  0.108 | 0.3999  0.5712  0.8458  0.5580  0.2237 | 0.4998  0.8230  0.9097  0.6975  0.4983 |

LCI = lower confidence limit, UCI = upper confidence limit, SE = standard error, P = p-value and FDR = false discovery rate. Models were adjusted for age.
